# Electric Drive Supervisor for Milling Process 4.0 Automation: A Process Analytical Approach with IIoT NIR Devices for Common Wheat

**DOI:** 10.3390/s20041147

**Published:** 2020-02-19

**Authors:** Silvia Grassi, Alessandra Marti, Davide Cascella, Sergio Casalino, Giuseppe Leonardo Cascella

**Affiliations:** 1 Department of Food, Environmental, and Nutritional Sciences (DeFENS), Università degli Studi di Milano, via G. Celoria 2, 20133 Milan, Italy; alessandra.marti@unimi.it; 2 GEM ICT Research & Development, Via Robert Schuman n.14, 70126 Bari, Italy; d.cascella@gemict.it; 3 Molino Casillo S.p.A., Via Sant’Elia Z.I., 70033 Corato, Bari, Italy; sergio.casalino@casillogroup.it; 4 Idea75 s.r.l., Via Brigata e Divisione Bari n.122, 70123 Bari, Italy; g.l.cascella@idea75.it

**Keywords:** common wheat, industry 4.0, NIR, PAT, PLS, quality by design, wheat quality

## Abstract

The milling industry envisions solutions to become fully compatible with the industry 4.0 technology where sensors interconnect devices, machines and processes. In this contest, the work presents an integrated solution merging a deeper understanding and control of the process due to real-time data collection by MicroNIR sensors (VIAVI, Santa Rosa, CA)—directly from the manufacturing process—and data analysis by Chemometrics. To the aim the sensors were positioned at wheat cleaning and at the flour blends phase and near infrared spectra (951–1608 nm) were collected online. Regression models were developed merging the spectra information with the results obtained by reference analyses, i.e., chemical composition and rheological properties of dough by Farinograph^®^ (Brabender GmbH and Co., Duisburg, Germany), Alveograph^®^ (Chopin, NG Villeneuve-la-Garenne Cedex, France) and Extensograph^®^.(Brabender GmbH and Co., Duisburg, Germany) The model performance was tested by an external dataset obtaining, for most of the parameters, R_PRED_ higher than 0.80 and Root Mean Squares Errors in prediction lower than two-fold the value of the reference method errors. The real-time implementation resulted in optimal (100% of samples) or really good (99.9%–80% of samples) prediction ability. The proposed work succeeded in the implementation of a process analytical approach with Industrial Internet of Things near infrared (IIoT NIR) devices for the prediction of relevant grain and flour characteristics of common wheat at the industrial level.

## 1. Introduction

In recent years, the needs along the value chain of wheat have changed. For each one of the many wheat uses, specific grain-quality requirements are preferred. The possibility to obtain reliable and quick information about kernels quality is constantly becoming more important for all the players of the wheat value chain. For instance, milling industry requires fast, simple and reliable methods for the control of grain quality during the reception phase, whereas the bread-making industry is searching for suitable methods able to predict the end-products quality. In the milling industry, quality control is of great importance for productivity maximization and standardization since wheat and flour have to respect specific compositional and functional requirements to satisfy customers’ requests. Indeed, the physico-chemical properties of the raw materials strongly affect the properties of the doughs during kneading, the consistency of the endproducts and the process efficiency. In this context, along decades, several approaches have been developed and proposed to assess quality along the chain. For instance, some approaches determine dough behavior during mixing (Farinograph^®^), providing information about the water absorption, the dough development time and its stability upon mixing [1]. Others predict dough resistance to a three dimensional (Alveograph^®^) or uniaxial (Extensograph^®^) force, providing information on dough strength and extensibility [2]. The indices obtained from the above-mentioned tests are useful to classify wheat (*Triticum aestivum* L.) in different quality categories, which correspond to flour end-uses. In the Italian context, common wheat flour is commercially classified in different categories, which range from the stronger to the weaker type, they are called improver wheat, superior bread making wheat, ordinary bread making wheat and wheat for biscuits [3]. The analyses involved in the determination of the rheological parameters are expensive, time consuming and require specialized personnel, thus there is concern over the development of alternative methods to be applied during the commercial transactions, when the products need to be characterized in very short times. Moreover, at present these quality parameters are controlled by the Quality by Test (QbT) approach, indeed, the quality is tested only on representative samples of the final product with the risk of having products below the desired quality threshold to be expensively reallocated.

This is in contrast with the modern Quality by Design (QbD) concept, based on the ability to monitor the process in real-time and accordingly adapts the production process to maximize product quality. In QbD, a quality profile is designed, and the production process is organized and controlled so to reach the target quality [4].

In the context of QbD, the Process Analytical Technologies (PAT) are the instruments allowing to evaluate process performances [5]. The evaluation is obtained with quantitative indexes regarding the improvements introduced by the planned process organization with the possibility of offering feedbacks to the control strategy of the process [6].

Spectroscopic sensors, in combination with multivariate data analysis, also known as Chemometrics, revealed to be successful as process analyzers in the food industry [7]. They take advantage of the interaction between the matter and the radiation—from different regions of the electromagnetic spectrum—to identify the chemical nature of molecules. In particular, spectroscopic instruments working in the near infrared region (NIR) are prompt to PAT application due to the rapidity of the measurement, a non-invasive character, the availability of miniaturized instruments and their resistance to harsh production environment (humid conditions, vibrations and dust presence) [5].

The challenges of QbD can be reached by exploiting the Industry 4.0 and Internet of Things (IoT) principles. The basic ideas of this new concept are based on the interconnection among machines, devices and sensors in the industry. The communication between different components of the industrial process allows sending control rules to the plant so to adapt the process behavior in real-time reaching the desired quality profile.

The capability to analyze the quality of the final product and to adapt to the production process in real-time is a breakthrough solution for the satisfaction of the Zero-Defect Manufacturing principles. This concept summarizes the potential of predicting unforeseen issues about the production policies or product characteristics, and avoids the loss of the final product, need for products reprocessing or complaints from the stakeholders involved in the wheat value chain.

Despite the great potential of near infrared spectroscopy (NIRS) and Chemometrics has been demonstrated for wheat quality characteristics [8,9,10] and rheological behavior [11,12,13,14] determination, most of the cited works deal with limited number of samples and/or laboratory scale approach, with a lack of real case studies about online/at-line process analysis at the industrial scale, as for many other food applications.

In this context, the study aims at filling the gap between the consolidated success of NIRS for cereal applications and the Zero-Defect Manufacturing, by the digital traceability along the production. To the aim, MicroNIR sensors (VIAVI, Santa Rosa, CA) were interfaced with the milling plant enabling the prediction of physico-chemical and rheological characteristics for wheat kernels and flour due to the development of robust regression models by the Partial Least Squares (PLS) algorithm. This will allow us to estimate ash, moisture, protein content, as well as dough rheological properties, on wheat kernel and flour directly on the plant, without the sampling procedure, thus to mix kernels and flours according to the desired quality characteristics of the final product.

The reader will find an explanation of the materials and methods used for the present industrial implementation in Section 2. In detail, the reference analyses, i.e., physico-chemical analysis and empiric rheological tests are presented in Section 2.1. Furthermore, the milling process and structure are schematically introduced together with the implementation of MicroNIR (VIAVI, Santa Rosa, CA) sensors (Section 2.2). The data analysis procedure and the used algorithm are presented in Section 2.3. The results structure will follow the same outline by presenting the results of the reference analysis and then the prediction capability of the developed models in term of the coefficient of determination (R^2^) and error (Root Mean Square Error) and compared with the Standard Error of Laboratory (SEL). Lastly, the results of real time implementation of the models will be presented in terms of prediction by comparing the actual data, including the SEL, with the value predicted by the PLS models.

## 2. Materials and Methods

Common wheat (*Triticum aestivum* L.) milling was monitored from October 2018 to August 2019 directly in the milling plant in two strategic points, i.e., at wheat cleaning (with the probe placed on a drop pipe before the wetting phase) and at flour blending phase, on the homogenization conveyor. For physico-chemical analysis and empiric rheological tests, conventionally used in the cereal chain, samples were collected from the process line at defined points ensuring the correspondence with the sample undergone to spectral analysis. Furthermore, NIR spectra were collected during the month of September 2019 to be used for the real-time validation of the IIoT NIR system.

### 2.1. Physico-chemical Analysis and Rheological Test

Ash content was determined by gravimetric analysis (ISO 2171:1993) [15], moisture content by moisture analyzer (ISO 712:2009) [16], protein content by Dumas method (FP628, LECO, Milan, Italy) [17], and gluten content by Perten Glutomatic (UNI EN ISO 21415-4:2007) [18]. Dough rheological properties were tested by Farinograph-E (Brabender GmbH and Co., Duisburg, Germany), Alveograph (Chopin, NG Villeneuve-la-Garenne Cedex, France) and Extensograph-E (Brabender GmbH and Co., Duisburg, Germany). 

By Farinograph analysis the mixing properties of the dough were determined by pasting 300 g of flour with water. The volume of water required to hydrate the flour to a predetermined value is adjusted according to the moisture content of the sample and then the amount of water required for the dough to reach a definite consistency (500 UB) is added according to the ISO 5530-1: 1997 approved method [19]. From the mixing profile from which the following indices were extrapolated: water absorption (Far-Abs) corresponding to the water per 100 g flour to reach the optimal consistency (500 Farinographic Units, FU) and expressed as g/100 g; water absorption at 14% (Far-Abs14%); dough development time (Far-Dev) defined as the interval from the first addition of water to the point in maximum consistency range immediately before the first indication of weakening and expressed in minutes; stability (Far-Stab) defined as the time difference between the point where the top curve first intersects 500-FU and the point where the top curve leaves 500-FU line and expressed in minutes and degree of softening (Far-DS) is the difference between the consistency value of the curve center at the end of the developing time and the curve centre 10 min after starting the test and it is expressed as FU.

The three-dimensional extension properties of dough were determined by an Alveograph analysis according to AACC 54-30.02: 1999 [20]. A thin sheet of dough is inflated by air pressure to form a bubble; by determining the pressure and length of time needed to burst the bubble, information about the dough tenacity, elasticity and baking strength can be obtained. From the raw profile the following indices were extrapolated: tenacity (Alv-P) corresponding to the maximum pressure on deformation and expressed in mm H_2_O; extensibility (Alv-L) corresponding to the length of the curve and expressed as mm; P/L ratio (Alv-P/L); strength (Alv-W) corresponding to the area under the curve and expressed in ×10^−4^ J; index of swelling (Alv-G) expressed in mm and elasticity index (Alv-Ie), which compares pressure after 200 mL volume of air have been blown into the dough test piece and expressed as percentage.

From the Extensograph-E (ISO 5530-2:2012) [21] dough extensibility was determined at 135 min. The analysis was performed with 300 g of flour mix with a sodium chloride solution (2.5% weight/volume), rounded and molded into a cylinder under constant conditions then it is left to rest at 30 °C. After that, the piece is extruded by a hook, which travels downwards at a constant rate, and the load on the dough piece is recorded, at the end of the test the consistency should arrive to 500 BU. Moreover, the Extensograph curve was obtained and the following indexes were determined: energy (Ext-En) corresponding to the area under the curve and expressed as cm^2^; extensibility (Ext-Ext) corresponding to the length of the curve and expressed as mm; resistance to extension (Ext-Res) related to the elastic properties and expressed as a Brabender Unit (BU) measured 50 mm after the curve has started and maximum resistance to extension (Ext-ResMAX) expressed as BU. 

### 2.2. Online MicroNIR Sensor Implementation and Data Acquisition 

**Figure 1:**
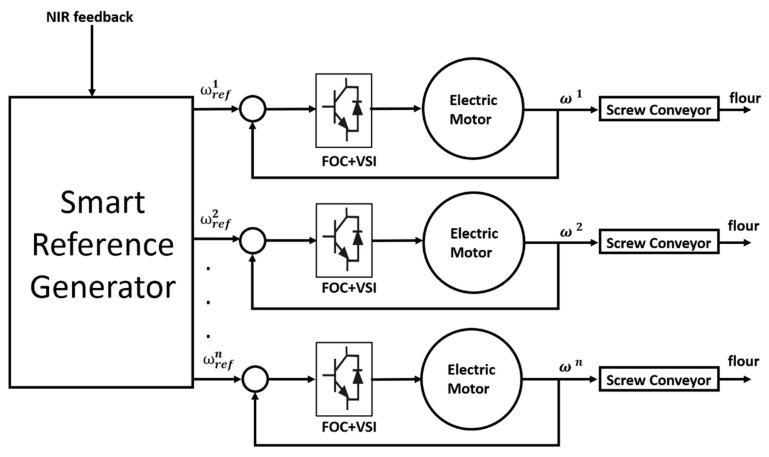
Overall milling process and structure. The speed references (ω_ref) are generated based on Near Infrared Spectroscopy (NIR) feedback and the electric motor is controlled using a Field-Oriented Control (FOC) and a Voltage Source Inverter (VSI). The speed of each motor is indicated with ω.

Figure 1 shows a scheme of the overall milling process and structure. A series of electric motors was controlled, each one actuating a screw conveyor. The speed reference was generated for each motor by the smart reference generator. The generator was a component programmed in a computer that produced the speed signal based on the NIR feedback. Finally, the inverter blocks were power converters typically used in electric drives to control electric motors.

In detail, a screw conveyor is the base of a typical milling process: by using a rotating helical screw installed within a tube or trough, the screw conveyor is responsible for the movement of the flour. The amount of volume transferred is proportional to the rotation of the screw. For this reason and since an electric motor actuates the conveyor, the motor itself must be controlled. The control is performed so to grant the correct flow of flour needed in the process. MicroNIR sensors (VIAVI, Santa Rosa, CA) were installed inside the screw conveyor structure so to record and analyze wheat data in real-time in a non-destructive manner. 

The NIR spectra were collected for each sampling point using a MicroNIR PAT-W (VIAVI, Santa Rosa, CA) and a MicroNIR PAT-U (VIAVI, Santa Rosa, CA) directly interfaced with the milling plant. The spectra were acquired in the spectral range of 950–1650 nm in diffuse reflectance, every 60 s with 7.3 μs of integration time. The collection was performed on kernels and flours for a total of more than 5000 spectra, considering that for each sample, i.e., grain or flour sampled from the process line, 10 spectra were measured.

### 2.3. Data Analysis

The spectra collected from October 2018 to August 2019 were averaged on sample bases, i.e., averaging the 10 spectra corresponding to the same grain or flour removed from the process line for the reference analyses. The spectra dataset was transformed by smoothing (Savitzky-Golay, 3 wavelengths gap size) followed by first derivative (Savitzky-Golay, 3 wavelengths gap size and 2nd order polynomial) and mean center in order to highlight the spectral features covered by the characteristic broad bands present in the NIR signal, thus allowing one to solve better the information retained in this kind of data. The pretreated spectra were used as the independent variable, X, whereas, physico-chemical and rheological results were used as the dependent variable, Y, for the Partial Least Squares (PLS) regression model development. PLS is one of the most common multivariate algorithms for linear regression development in chemistry and technology. It well manages the problem of noise, collinearity and large number of variables, thus, being ideal for NIR data. Indeed, PLS returns a model of X as a bilinear projection considering that the information retained in X is uncorrelated to the Y solving noise and irregularities of X and not related to Y. In detail, the totality of the data was randomly split into a calibration dataset to be used for model development and a validation dataset to test the prediction capability for both kernel and flour samples.

The number of samples used for calibration differs for each PLS model as not all the destructive analyses were performed on all the samples due to complexity and time required for their execution; however at least 90 samples/objects were used for this purpose.

A regression model was calibrated for each physico-chemical compound or empiric rheological feature, i.e., each single parameter determined with the conventional methods was considered separately as a dependent variable for the construction of the calibration models. The models were cross-validated by venetian blind to choose the optimal number of latent variables (LVs) by minimizing the error in cross-validation and then tested in prediction by the validation dataset.

The performance of the models in calibration, cross-validation and prediction was evaluated in term of coefficient of determination, R^2^, and Root Mean Square Error (RMSE), which reports the error of the PLS model expressed in the same units of the dependent variable. Furthermore, the prediction capability was evaluated by comparison of the predicted value for each parameter and the laboratory reproducibility, i.e., coefficient of variation (CV) or Standard Error of Laboratory (SEL).

The prediction ability of the developed models was tested online with the sampling occurred on September 2019. This further test was performed to consider the model robustness with different type of kernels and flour and in a different season. The sampling was performed as previously described, and then the new spectra were used as an independent test set. The predicted values of these new data by the previously developed models were compared with the measured characteristics. The performance of the model was evaluated in terms of prediction by comparing the actual data, including the laboratory error, with the value predicted by the PLS model.

All the data analyses were performed in Unscrambler X software version 10.4 (CAMO, Trondheim, Norway). 

## 3. Results and Discussion

### 3.1. Kernel and Flour Characterisation by Conventional Techniques 

#### 3.1.1. Chemical Composition

The chemical composition of the samples is summarized in Table 1. The moisture content of the samples was in the range required by the Italian law [22], with a maximum content of 15.5%. The ash content ranged from 0.5 to 1.72 g/100 g, suggesting that the selected samples belonged to a wide range of extraction rate (from refined flour to whole meal flour). According to the Italian law [22] the refined flour must have a maximum level of ash of 0.55 g/100 g whereas the ash content in the whole meal flour has to range between 1.3 and 1.7 g/100 g. As regards the protein content, the values were above 9.00 g/100 g, which is the minimum values required according to the Italian law. Based on the Italian wheat classification, the protein content—together with protein quality—is related to the end-use of the flour. Specifically, flours with a protein content below 11% are used for biscuit or pastry, flours with a protein content between 11 and 13 are used for bread, whereas flours with protein content higher than 14% are used to produce baked products that require long fermentation time. Finally, the gluten content of the samples ranged from 7.10 to 14.50 g/100 g. Neither the Italian law nor the classification includes the gluten content as an index of quality. On the contrary, dough rheology is used to define wheat quality (see paragraph below). For all the indices here considered, the SEL values ranged from 3% and 7%, for gluten and protein content, respectively.

#### 3.1.2. Rheological Properties

Data from the main rheological tests carried out on either kernels or refined wheat flours are reported in Table 2.

The Farinograph test is widely used to measure dough behavior during mixing (i.e., water and time required to make a dough, and its stability during mixing), whereas, the Alveograph test is widely used to measure the tenacity (Alv-P), strength (Alv-W) and the extensibility (Alv-L) of a dough subjected to a 3D-extension of the dough due to the insufflation of air. On the contrary, the dough resistance to a uniaxial extension is given by the Extensograph test.

According to the Italian wheat classification, improved wheats are characterized by W ≥ 300 × 10^−4^ J, a P/L between 0.6 and 1 and a farinographic stability (Far-Stab) ≥ 15 min; superior bread making wheats have W between 160 and 220 × 10^−4^ J, P/L ≤ 0.6 and stability between 10 and 15 min. In addition, wheats with W 115 and 160 ×10^−4^ J, P/L ≤ 0.6 and stability between 5 and 10 min are classified as ordinary bread making wheat. Finally, wheat flours with W ≤ 115 × 10^−4^ J, P/L ≤ 0.5 and stability < 5 min are used for biscuits [4]. The samples considered cover the whole variability of the parameters defined by the Italian law, also exceeding the classification ranges as to represent the possible variability of raw materials managed by the milling plant, thus providing a more robust starting point for the regression model development.

### 3.2. Kernel and Flour Characterisation by Near Infrared Spectroscopy 

**Figure 2:**
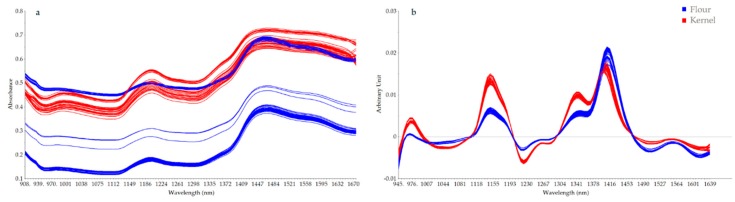
Spectra acquired for the kernels (red) and for the flour (blue): (**a**) raw spectra and (**b**) spectra after smoothing (Savitzky-Golay, 3 wavelengths gap size) and first derivative (Savitzky-Golay, 3 wavelengths gap size and 2nd order polynomial) transformation.

Spectral features observed along the processes confirmed what previously reported. Indeed, Juhász et al. (2005) [23] and Workman and Weyer (2012) [24] attributed features in the regions between 1154–1166 and 1456–1472 nm related to moisture content and the signal between 1578 and 1582 nm to starch absorption. Similar spectra profiles were observed for the spectra collected for kernel and flour even though the kernel spectra are characterized by higher absorbance Figure 2a, which remains visible in the profiles after smoothing (Savitzky-Golay, 3 wavelengths gap size) and first derivative transformation (Savitzky-Golay, 3 wavelengths gap size and 2nd order polynomial) Figure 2b.

The expected difference is related to both physical effect (different kernel size and flour particle size) and chemical characteristics of the two products, mainly water content. However, it is difficult to associate bands in the considered region of NIR spectra for samples so rich in moisture to specific vibrations; clearly a multivariate approach is needed to uncover the information hidden in the broad band characterizing the NIR spectra.

### 3.3. PLS Regression Models for Kernel and Flour Characterisation

The results of the PLS models for the 18 reference parameters are reported in Table 3 in terms of samples used to calibrate and validate the models, model dimensionality (i.e., number of selected latent variables, LVs), RMSE and R^2^ or R statistics. The representativeness of the validation set has been guaranteed as the maximum and minimum values of each parameter fall into the range established by the calibration set.

Thirteen out of eighteen models reached R_PRED_ higher than 0.80 and RMSEP lower or comparable to the CV of the reference analysis. As expected, models developed for composition parameters (ash, moisture and protein) obtained the highest R_PRED_, being 0.96, 0.98 and 0.93, respectively.

Wheat protein content has been one of the major targets for PLS models development from NIR data reaching optimal coefficient of determination and RMSE in prediction in different studies such as 0.97 and 0.31 g/100 g, respectively [25]. Even though in our study the two figure of merits were slightly lower, it should be considered that the number of samples used to build the model by Shi and Peiqiang (2017) [25] was lower (*n* = 80) than ours (*n* = 223); furthermore their study was performed by benchtop instrument analyzing the whole Vis-NIR range (680–2500 nm) and at laboratory scale. Similar consideration could be done for moisture and ash. Indeed, Dong and Sun (2013) [26] obtained a moisture predictive model with RMSEP of 0.088 g/100 g and a R_PRED_ of 0.98 and an ash predictive model with RMSEP of 0.019 g/100 g and a R_PRED_ of 0.91. As far as concern gluten, PLS models developed by different research groups led to controversial results, however high performances were obtained in prediction RMSEP of 0.81 g/100 g and a R^2^_PRED_ of 0.98 [27], close to our validation performances.

The model prediction ability for Far-Abs is in line with literature survay, which also in this case considered mainly laboratory scale studies. Indeed, the resulting RMSEP (1.21 g/100 g) is included in the 1.0–1.6 g/100 g range [11,12]. Far-Stab models gave higher error in prediction (RMSEP of 6.24 min) when compare to literature results (around 1 min of error in prediction, SEP) [13], however, the mentioned authors worked with few samples (*n* = 79) at the laboratory scale.

The degree of softening, Far-DS, has been generally not well predicted in previous studies such as the models reported by Chen et al. (2017) [11] having an RMSEP of 33 FU; whereas our result (RMSEP of 13.57 FU) is in line with Mutlu et al.’s (2011) [13] findings (RMSEP of 11 FU).

PLS models developed for the indexes extrapolated from the alveographic analysis gave reliable results with the exception of Alv-P/L, however the poor P/L regression curve is not even a relevant issue as the single parameters, Alv-P and Alv-L could be predicted by the specific PLS models for then calculating their ratio, thus skipping the modeling failure.

The model developed for W gave really good results, being the error in prediction of 28.67 × 10^−4^ J in line with a previous work [12] or even lower with respect to Dowell et al. (2006) [14]. The performance of the model has a quite relevant impact in the cereal industry as it is related to flour strength, generally considered to define the technological behavior of flours and their end use. Good results were also obtained for Alv-G and Alv-Ie, however it is difficult to compare with previous findings as, in both cases, only one study developed a PLS model. Moreover, the model developed for Alv-G was not tested in prediction [14] and the Alv-Ie model did not give reliable results [13]. 

Regarding the PLS models developed from the Extensograph analysis, the Ext-En and Ext-Ext models gave good results, with low error and high R, however the Ext-ResMAX was not that reliable in terms of R, being the lowest value obtained, and error as well.

To establish the effectiveness of the prediction ability it is relevant to compare the regression models’ error with the errors of the reference methods. According to Shenk and Westerhaus (1996) [28] if the SEP is less than two times the value of reference method error (SEL), the model’s performance in terms of error in prediction should be considered an excellent estimation. In Table 4 are reported the minimum and maximum SEL for each parameter and the 2SEL, i.e., the threshold below which the SEP should be to have an excellent estimation by the PLS models. The models tested for moisture, protein, Far-DS, Alv-G, Ext-En and Ext-Ext are characterized by SEP lower than the 2SEL calculated for the minimum value of each considered parameter. All the other PLS models predicted each parameter with a SEP lower or equal to the maximum 2SEP, except for Far-Abs 14% and dough development time (Far-Dev) Table 4.

### 3.4. Online Testing of the Prediction Ability of the PLS Models 

The models developed in the first phase, i.e., using the data acquired from October 2018 to August 2019, were tested for real-time prediction. In this way, the underlying idea is to exploit the PLS model prediction to generate the reference signal sent to the motor so to adapt the process behavior based on the real-time wheat and flour characteristics and the desired quality of the product. In other words, the desired speed of the motor is automatically adjusted in order to meet the production requirements.

The predicted values for all the developed models were compared with the measured characteristics. The performance of the model was evaluated in terms of prediction by comparing the actual data, including the laboratory error, with the value predicted by the PLS model. Table 5 summarizes the prediction ability for wheat kernels and flour for all the evaluated parameters.

The models that obtained more than 100% of correctly predicted samples were judged as optimal, from 99.9 to 80 really good, from 79.9 to 70 good, from 70 to 50 fairly good and below 50 scarce. From Table 5 it is possible to understand that all the models gave optimal or really good prediction ability when used in prediction on kernels, with the exception of Far-Stab and Far-DS. P/L index was not considered due to the scarce prediction ability obtained for the models in the previous steps and the possibility of calculating the ratio from the P and L prediction.

On the other hand, the real-time testing for flour data had poorer performances when considering the prediction of farinographic results, whereas for the other parameters the real-time monitoring gave promising results for the industrial implementation. For the future it is envisioned the inclusion or new flour data in the calibration set to increase the variability of flour especially in terms of farinographic indexes. 

## 4. Conclusions

In the described process, the interconnection between milling plants and the outputs of the MicroNIR sensors made possible the automatic control of the electric motors in real-time, avoiding the need for a manual tune of the screw conveyor behavior and the motor controller. Moreover, the interconnection between devices allowed for coordinating each motor. In this way, higher productivity and quality was reached when compared to a non-automatic and non-interconnected control of the drives. The developed models guaranteed the prediction of chemical composition and rheological properties of dough (indexes extrapolated from Farinograph®, Alveograph® and Extensograph®) with R_PRED_ higher than 0.80 and RMSEP lower than two-fold the value of the reference method errors. Furthermore, the real-time implementation resulted in optimal (100% of samples) or really good (99.9%–80% of samples) prediction ability.

The presented idea is an innovative solution for the milling process control since it exploits the potentialities offered by Industry 4.0 where sensors interconnect devices, machines and processes. The result was optimal management of the production process that can successfully predict different product characteristics and automatically adapt itself to grant the desired level of quality to the product. In accordance with the principle of Zero-Defect Manufacturing, the outcome was the minimization of the probability of product failures and complaints from customers. Indeed, in the milling industry the bland of flours/kernels with specific properties was the key point in order to obtain final product respecting the quality characteristics required by costumers according to the end use of the product itself. Due to the proposed approach—combining NIR spectroscopy, Chemometrics and the integration of intelligent systems—the process parameters could be controlled in real time. Thus, unforeseen issues about the production policies or product characteristics could be predicted, and the loss of the final product could be avoided.

Even though a robust sampling plan was performed along an entire year, a future perspective could be the transfer of the developed models to other milling plants, thus creating an IoT not just along one transformation chain but horizontally along more production lines. Together with that, NIR sensors could be positioned at other strategic plant levels, such as at raw materials delivery allowing the kernels control for characteristic parameters.

## Figures and Tables

**Table 1:** Chemical parameters of the analyzed samples.

Parameter	N	Min	Max	Mean	Median	SEL
Ash (g/100 g)	513	0.50	1.72	0.75	0.58	5
Moisture (g/100 g)	511	11.2	15.50	14.33	14.90	5
Protein (g/100 g)	364	9.71	15.53	11.59	11.03	7
Gluten (g/100 g)	169	7.10	14.50	9.54	9.00	3

Number of analyzed samples (N), Standard Error of Laboratory (SEL).

**Table 2:** Rheological properties of the analyzed samples.

Parameter	N	Min	Max	Mean	Median	CV%	SEL
Far-Abs (g/100 g)	210	16.70	64.70	54.26	54	1	
Far-Abs14% (g/100 g)	208	49.30	58.00	54.31	53.95	1	
Far-Dev (min)	162	1.00	59.20	11.84	1.7	-	< 3 min: ± 0.5 min 3–6 min: ± 1 min > 6 min: ± 2 min
Far-Stab (min)	174	1.40	37.90	8.50	5.2	-	< 4 min: ± 1 min 4–10 min: ± 2 min 10–20 min: ± 3 min > 20 min: ± 4 min
Far-DS (FU)	185	2.00	128.00	60.89	63.00	-	< 100 FU: ± 20 FU > 100 FU: ± 25 FU
Alv-P (mm)	153	43.00	100.00	71.20	73.00	8	
Alv-L (mm)	242	41.00	138.00	92.25	94.00	8	
Alv-P/L	232	0.34	2.41	0.82	0.70	8	
Alv-W [* 10^−4^ J]	243	112.00	435.00	217.62	201.00	8	
Alv-G (mm)	210	14.30	26.10	21.40	21.60	5	
Alv-Ie [%]	283	49.0	64.30	57.05	56.90	-	-
Ext-En [cm^2^]	155	59.00	158.00	93.46	90.00	-	<300 BU: ± 10 300–500 BU: ± 15 > 500 BU: ± 20
Ext-Ext (mm)	144	113.00	216.00	157.12	152.00	-	<300 BU: ± 10 mm 300–500 BU: ± 10 mm > 500 BU: ± 15 mm
Ext-ResMAX [BU]	154	159.00	691.00	419.88	428.00	10	

Coefficient of Variation (CV); Standard Error of Laboratory (SEL); Water Absorption (Far-Abs), Water Absorption at 14% (Far-Abs14%); dough Development time (Far-Dev); Stability (Far-Stab); Degree of Softening (Far-DS); tenacity (Alv-P); extensibility (Alv-L); P/L ratio (Alv-P/L); strength (Alv-W); index of swelling (Alv-G); elasticity index (Alv-Ie); Energy (Ext-En); Extensibility (Ext-Ext); Maximum Resistance to extension (Ext-ResMAX); Brabender Unit (BU).

**Table 3:** Results of the Partial Least Squares (PLS) models of the 18 reference parameters calculated from the MicroNIR spectra acquired from October 2018 to August 2019.

		Calibration					CV		Prediction					
Parameter	LVs	N	Min	Max	RMSE	R^2^	RMSE	R^2^	N	Min	Max	RMSE	SEP	R
Ash (g/100 g)	5	350	0.50	1.72	0.07	0.95	0.08	0.95	163	0.51	1.66	0.17	0.17	0.96
Moisture (g/100 g)	4	346	11.20	15.40	0.19	0.97	0.20	0.97	165	11.20	14.13	0.32	0.32	0.98
Protein (g/100 g)	7	223	9.71	15.53	0.32	0.94	0.37	0.93	141	10.70	11.09	0.47	0.45	0.93
Gluten (g/100 g)	9	118	7.10	14.50	0.34	0.94	0.34	0.91	51	9.40	13.10	0.79	0.72	0.87
Far-Abs (g/100 g)	8	133	50.10	60.90	0.63	0.90	0.70	0.88	77	50.20	60.00	1.21	1.19	0.81
Far-Abs14% (g/100 g)	8	138	51.00	59.70	0.61	0.85	0.67	0.83	70	51.00	59.50	1.25	1.19	0.70
Far-Dev (min)	6	97	1.00	59.20	2.09	0.99	2.39	0.99	67	1.00	58.50	6.24	6.07	0.95
Far-Stab (min)	8	91	1.70	37.90	1.52	0.98	1.93	0.96	71	1.50	36.20	2.88	2.60	0.97
Far-DS (FU)	6	107	2.00	128.00	7.54	0.95	8.16	0.94	68	4.00	124.00	13.57	13.65	0.92
Alv-P (mm)	12	99	43.00	100.00	4.39	0.90	5.12	0.87	54	45.00	100.00	10.17	10.27	0.79
Alv-L (mm)	8	176	41.00	136.00	11.10	0.76	11.97	0.72	66	46.00	138.00	13.24	13.30	0.81
Alv-P/L	10	166	0.34	2.40	0.22	0.69	0.23	0.64	66	0.35	2.23	0.34	0.28	0.64
Alv-W (×10^−4^ J)	9	178	112.00	435.00	22.06	0.89	23.55	0.87	65	114.00	364.00	29.30	28.67	0.90
Alv-G (mm)	9	147	14.30	26.00	1.14	0.81	1.28	0.76	63	15.10	26.10	1.38	1.38	0.86
Alv-Ie (%)	7	198	49.30	69.30	1.54	0.79	1.64	0.76	85	49.40	65.00	2.62	2.53	0.71
Ext-En (cm^2^)	7	99	160.00	59.00	8.00	0.87	8.98	0.84	56	60.00	158.00	11.60	11.70	0.88
Ext-Ext (mm)	8	90	113.00	216.00	6.94	0.88	7.95	0.84	54	116.00	213.00	12.21	12.11	0.81
Ext-ResMAX (BU)	7	98	161.00	691.00	35.92	0.90	40.52	0.86	56	162.00	659.00	93.74	94.51	0.62

Cross-validation (CV); Minimum (Min); Maximum (Max); Latent Variables (LVs); Number of samples used to construct or validate the model (N); Root Mean Square Error (RMSE); Coefficient of determination (R^2^); Standard Error of Prediction (SEP); Water Absorption (Far-Abs), Water Absorption at 14% (Far-Abs14%); dough Development time (Far-Dev); Stability (Far-Stab); Degree of Softening (Far-DS); tenacity (Alv-P); extensibility (Alv-L); P/L ratio (Alv-P/L); strength (Alv-W); index of swelling (Alv-G); elasticity index (Alv-Ie); Energy (Ext-En); Extensibility (Ext-Ext); Maximum Resistance to extension (Ext-ResMAX); Brabender Unit (BU).

**Table 4:** Comparison of the Standard Error of Laboratory (SEL) and the Standard Error of Prediction (SEP) resulting from the PLS models of the 18 reference parameters calculated from the MicroNIR spectra acquired from October 2018 to August 2019.

	**Chemical** **(g/100 g)**				**Farinograph**				
	**Ash**	**Moisture**	**Protein**	**Gluten**	**Abs (g/100 g)**	**Abs14% (g/100 g)**	**Dev (min)**	**Stab (min)**	**DS (FU)**
SEL min	0.03	0.56	0.68	0.21	0.17	0.49	0.50	1.00	20.00
SEL max	0.09	0.78	1.09	0.44	0.65	0.58	2.00	4.00	25.00
2 × SEL min	0.05	1.12	1.36	0.43	0.33	0.99	1.00	2.00	40.00
2 × SEL max	0.17	1.55	2.17	0.87	1.29	1.16	4.00	8.00	50.00
SEP	**0.17**	**0.32**	**0.45**	**0.72**	**1.19**	1.19	6.07	**2.60**	**13.65**
	**Alveograph**						**Extensograph**		
	**P (mm)**	**L (mm)**	**P/L**	**W (10^−4^ J)**	**G (mm)**	**Ie (%)**	**En (cm^2^)**	**Ext (mm)**	**ResMAX (BU)**
SEL min	2.40	3.28	0.03	2.88	0.72	-	10.00	10.00	4.70
SEL max	8.80	12.00	0.19	34.80	1.37	-	20.00	15.00	69.10
2 × SEL min	4.80	6.56	0.05	5.76	1.43	-	20.00	20.00	9.40
2 × SEL max	17.60	24.00	0.39	69.60	2.73	-	40.00	30.00	138.2
SEP	**10.27**	**13.30**	**0.28**	**28.67**	**1.38**	2.53	**11.70**	**12.11**	94.51

Standard Error of Laboratory (SEL); Standard Error of Prediction (SEP); Water Absorption (Far-Abs), Water Absorption at 14% (Far-Abs14%); dough Development time (Far-Dev); Stability (Far-Stab); Degree of Softening (Far-DS); tenacity (Alv-P); extensibility (Alv-L); P/L ratio (Alv-P/L); strength (Alv-W); index of swelling (Alv-G); elasticity index (Alv-Ie); Energy (Ext-En); Extensibility (Ext-Ext); Maximum Resistance to extension (Ext-ResMAX); Brabender Unit (BU).

**Table 5:** Online performance of the calibrated models: percentage of correct prediction for the real-time testing of the developed models in case of kernels and flour probe.

Parameter	Kernel	Evaluation	Flour	Evaluation
Ash	100 (50)	optimal	100 (82)	optimal
Moisture	100 (50)	optimal	100 (126)	optimal
Protein	94 (50)	really good	100 (108)	optimal
Gluten	92 (50)	really good	-	-
Far-Abs	80 (30)	really good	13 (50)	scarce
Far-Abs14%	83 (30)	really good	24 (50)	scarce
Far-Dev	100 (50)	optimal	11 (61)	scarce
Far-Stab	72 (50)	good	39 (61)	scarce
Far-DS	62 (50)	fairly good	11 (61)	scarce
Alv-P	85 (20)	really good	84 (72)	really good
Alv-L	95 (20)	really good	88 (72)	really good
Alv-P/L	-	-	-	-
Alv-W	100 (20)	optimal	57 (72)	fairly good
Alv-G	100 (20)	optimal	92 (72)	really good
Alv-Ie	100 (20)	optimal	96 (72)	really good
Ext-En	86 (21)	really good	76 (25)	good
Ext-Ext	100 (21)	optimal	76 (25)	good
Ext-ResMAX	95 (20)	really good	88 (25)	really good

Correct prediction expressed as percentage. In brackets the numerosity of samples tested is reported.
